# [Retracted] *Celastrus orbiculatus* extracts induce apoptosis and inhibit invasion by targeting the maspin gene in human gastric adenocarcinoma cells

**DOI:** 10.3892/ol.2023.13919

**Published:** 2023-06-19

**Authors:** Yayun Qian, Songhua Lu, Youyang Shi, Xueyu Zhao, Ting Yang, Feng Jin, Yanqing Liu

Oncol Lett 15: 243–249, 2018; DOI: 10.3892/ol.2017.7341

Following the publication of this paper, it was drawn to the Editors’ attention by a concerned reader that, in the cellular images shown in Fig. 1D on p. 245, a pair of data panels appeared to be strikingly similar, such that these data were likely to have been derived from the same original source even though they were intended to show the results from differently performed experiments; similarly, multiple instances of overlapping data panels were identified with the invasion and migration assay experiments shown in Fig. 3 on p. 247.

Owing to an overall lack of confidence in the presented data, the Editor of *Oncology Letters* has decided that this paper should be retracted from the Journal. After contacting the authors, they accepted the decision to retract this paper. The Editor apologizes to the readership for any inconvenience caused.

